# Efficient, compact, and versatile: Type I‐F2 CRISPR‐Cas system

**DOI:** 10.1002/mlf2.12145

**Published:** 2024-09-22

**Authors:** Shengsheng Ma, Senfeng Zhang, Kunming Liu, Tao Hu, Chunyi Hu

**Affiliations:** ^1^ Department of Biological Sciences, Faculty of Science National University of Singapore Singapore Singapore; ^2^ Department of Biochemistry, Yong Loo Lin School of Medicine National University of Singapore Singapore Singapore; ^3^ Precision Medicine Translational Research Programme (TRP) National University of Singapore Singapore Singapore

Type I CRISPR‐Cas systems are the most prevalent and versatile among CRISPR‐Cas systems, widely distributed across prokaryotes^1^. These systems are composed of multi‐subunit complexes, known as Cascade (CRISPR‐associated complex for antiviral defense), which function by binding to CRISPR RNAs (crRNAs) and targeting complementary DNA sequences for degradation. Type I CRISPR‐Cas systems, including subtypes I‐A to I‐G, have been extensively studied for their potential in genome manipulation^2^, ^3^, ^4^, ^5^, ^6^, ^7^, ^8^, ^9^, ^10^, ^11^, ^12^.

**Figure 1:**
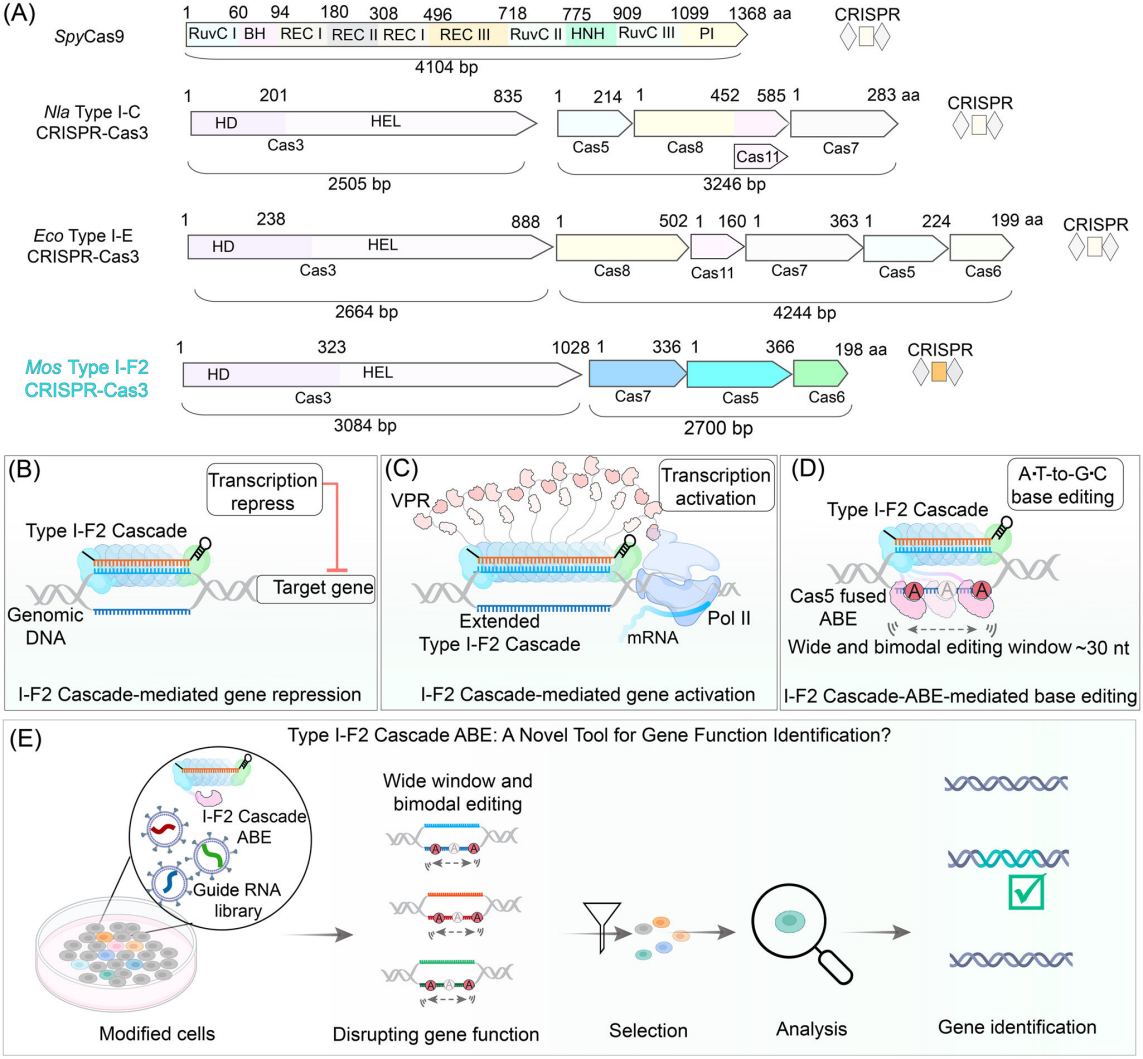
Overview of the Mos350 type I‐F2 CRISPR‐Cas system and its applications. (A) Comparison of different CRISPR‐Cas systems, highlighting the compact size of the Cascade complex from Mos350 Type I‐F2 CRISPR‐Cas3 system. The diagram includes *Spy*Cas9 (4104 bp), *Nla* Type I‐C CRISPR‐Cascade (3246 bp), *Eco* Type I‐E CRISPR‐Cascade (4244 bp), and *Mos* Type I‐F2 CRISPR‐Cas3 (2700 bp). The Mos350 Type I‐F2 Cascade comprises only three subunits (Cas7, Cas5, and Cas6) with a total gene size of 2700 bp, making it significantly smaller than other systems. (B) Schematic of Type I‐F2 Cascade‐mediated gene repression in genomic DNA. The Cascade complex targets and represses the expression of the target gene by binding to its promoter region. (C) Schematic of extended Type I‐F2 Cascade‐mediated gene activation in human cells. The Cascade complex, fused with the VPR (VP64‐p65‐Rta) transcriptional activation domain, activates gene expression by recruiting the transcription machinery to the target site. (D) Illustration of Type I‐F2 Cascade‐ABE (Adenine Base Editor)‐mediated base editing. The Cas5 subunit is fused with the ABE enzyme, enabling A‐to‐G base editing with a wide and bimodal editing window of approximately 30 nucleotides. (E) Proposed application of the Type I‐F2 Cascade ABE as a novel tool for gene function identification. A guide RNA library is used to disrupt gene function across a population of modified cells. The cells undergo selection based on functional or phenotypic outcomes, followed by analysis to identify the disrupted genes, demonstrating the utility of the wide and bimodal editing window in functional genomic screens. *Eco*, *Escherichia coli*; *Mos*, *Moraxella osloensis; Nla, Neisseria lactamica*.

Although Type I CRISPR‐Cas systems offer significant potential for genome editing and transcriptional regulation, they face notable difficulties when used in eukaryotic cells. The main challenge stems from their considerable size and intricate multi‐subunit architecture, which complicate their delivery especially when using viral vectors with strict cargo size limits, such as adeno‐associated viruses (AAVs). For example, the Cascade complex of the well‐studied *Escherichia coli* Type I‐E CRISPR‐Cas3 system consists of five subunits with a total gene size exceeding 4.2 kb^7^. Even the more compact Type I‐C Cascade from *Neisseria lactamica* still comprises four subunits, over 3.2 kb in gene size^13^, ^14^ (Figure 1A), which is challenging to package and deliver effectively. Moreover, the assembly of these multiple subunits within cells can be inefficient, leading to reduced activity. Furthermore, the lack of a universal protospacer adjacent motif (PAM) across different Type I CRISPR‐Cas systems adds another layer of difficulty, as it limits the range of targetable sequences. These limitations highlight the need for more compact and efficient CRISPR systems, particularly for therapeutic applications that require precise and reliable gene editing.

The latest study published in *Nature Communications* presents a milestone in genome editing and transcriptional regulation within human cells, leveraging the smallest Type I system known to date, the I‐F2 subtype^15^. Guo et al. focused on overcoming the limitations of traditional Type I CRISPR‐Cas systems by developing a minimal version of the I‐F2 subtype, derived from *Moraxella osloensis* CCUG 350. The I‐F2 subtype is the most compact among Type I systems, consisting of only three essential subunits—Cas5, Cas6, and Cas7—with a total gene size of approximately 2.7 kb, making it significantly smaller than the widely used SpyCas9 system (Figure 1A). The researchers began by screening 93 different Type I‐F2 systems from various bacterial species to identify the most compact and functional systems. After extensive analysis, they identified the I‐F2 system from *M. osloensis* as the most promising candidate due to its small size, simple 5′‐CC PAM requirement, and high activity in human cells.

The authors began their investigation by first testing the efficacy of Type I‐F2 CRISPR‐Cas systems in *E. coli*. They screened eight different I‐F2 systems by expressing the Cascade complex and using it to repress the expression of a *GFP* reporter gene (Figure 1B). This screening allowed them to identify which systems were capable of forming functional Cascade complexes for efficient DNA targeting in prokaryotic cells. Following this, they extended their screening to human cells, where they evaluated the expression, assembly, and crRNA processing of 11 different I‐F2 systems. Of these, five systems demonstrated robust expression and effective crRNA processing, making them suitable candidates for further experimentation. Among the five, the I‐F2 system derived from *M. osloensis* CCUG 350 (Mos350) was selected for its superior performance. The researchers then focused on engineering the Mos350 I‐F2 system for transcriptional activation in human cells. They explored different fusion strategies by attaching the VPR (VP64‐p65‐Rta) transcriptional activation domain to various subunits of the Cascade complex. Through these experiments, they found that fusing VPR to the Cas7 subunit yielded the most potent transcriptional activation, significantly outperforming other configurations (Figure 1C).

To further enhance the system's performance, the team engineered crRNAs with varying spacer lengths, discovering that longer spacers, such as 50 nucleotides, significantly improved transcriptional activation efficiency. They then optimized the Cas7 subunit of the Cascade by introducing specific mutations, such as L175F, further enhancing the system's activity. These cumulative improvements resulted in a version of the Mos350 I‐F2 system that not only matched but surpassed the transcriptional activation capabilities of the widely used dCas9‐VPR system at certain loci.

Building on these successes, the authors then explored the potential of the Mos350 I‐F2 system as a base editor by fusing adenine deaminase (ABE) to different Cascade subunits. After extensive testing, they identified the 5NABE configuration—where the deaminase was fused to the N‐terminus of Cas5—as the most effective. This base editor exhibited a highly efficient and broad editing window, characterized by a bimodal distribution of activity, enabling precise edits across a wide range of target sites (Figure 1D). The authors also performed off‐target analyses, confirming the system's high specificity and minimal off‐target effects. Through these well‐designed experiments, the authors demonstrated the versatility and efficiency of the Mos350 I‐F2 CRISPR‐Cas system, establishing it as a powerful tool for both transcriptional activation and precise genome editing (Figure 1B–D).

Looking ahead, one of the primary areas for future exploration is testing the I‐F2 CRISPR‐Cas system across various cell lines beyond the HEK293T cells used in this study. Expanding this research into other mammalian cells, including primary cells and stem cells, will be crucial to understanding the broader applicability and efficiency of the system in different biological contexts. Another significant avenue is optimizing delivery methods. While this study utilized plasmid‐based delivery, future work should explore alternative delivery strategies, such as adeno‐associated virus (AAV) vectors or lipid nanoparticle‐encapsulated mRNA. These methods might offer more efficient, tissue‐specific, and clinically relevant delivery, particularly for therapeutic applications.

The broad editing window of the I‐F2 ABE, spanning up to 30 nucleotides with a bimodal distribution^15^, presents a unique opportunity for developing novel CRISPR screening techniques. By utilizing a guide RNA library and extensive base editing to induce random gene knockouts, researchers could perform functional and phenotypic screenings to identify gene functions, offering a powerful tool for genetic research and drug discovery (Figure 1E). However, while the broad editing window is advantageous for gene disruption, there is also a critical need to refine and narrow this window for specific applications, particularly in medical therapeutics. Enhancing the precision of the editing window could lead to safer and more targeted gene therapies, reducing the risk of unintended mutations and improving the overall safety profile of CRISPR‐based treatments. Additionally, while the efficiency of the I‐F2ABE editor is significantly higher than that of Cas12‐derived ABE editors, it remains lower compared to Cas9‐based ABE editors. Therefore, future efforts could also focus on engineering this system to further improve its base editing efficiency.

In summary, the versatility of the I‐F2 CRISPR‐Cas system offers immense potential, with future research poised to both expand its applicability and refine its precision, paving the way for innovative applications in both basic research and clinical settings.
